# Optimal Maintenance Strategy for Patients with Improved Left Ventricular Function Following Sacubitril/Valsartan Therapy

**DOI:** 10.3390/medicina61081487

**Published:** 2025-08-19

**Authors:** Yoonjee Park, Minjung Bak, Heayoung Shin, David Hong, Jeong Hoon Yang, Darae Kim, Eun-Seok Jeon, Jin-Oh Choi

**Affiliations:** 1 Department of Cardiology, Bucheon Sejong Hospital, 28, Bucheon-si 14754, Republic of Korea; 2 Division of Cardiology, Department of Medicine, Heart Vascular Stroke Institute, Samsung Medical Center, Sungkyunkwan University School of Medicine, 81 Irwon-ro, Gangnam-gu, Seoul 06351, Republic of Korea; 3 Division of Cardiology, Department of Internal Medicine, Korea University Guro Hospital, Korea University, Seoul 06351, Republic of Korea

**Keywords:** heart failure, ejection fraction, sacubitril/valsartan, N-terminal-pro hormone B-type natriuretic peptide

## Abstract

*Background and Objectives*: Optimal pharmacological treatment following left ventricular ejection fraction (LVEF) improvement remains largely unknown. This study compared the clinical outcomes of patients with heart failure (HF) with improved EF (HFimpEF) based on the maintenance of sacubitril/valsartan (S/V) or transition to a renin–angiotensin system blocker (RASB). *Material and Method*: A total of 354 patients with recovered LVEF of at least 40% after S/V treatment from a single center were retrospectively analyzed. Patients were categorized into three groups: those who continued S/V (*n* = 294), those who switched to RASB (*n* = 47), and those who discontinued both S/V and RASB (*n* = 13). The primary endpoint was HF relapse, defined as a two-fold increase in baseline serum N-terminal-pro hormone B-type natriuretic peptide (NT-proBNP) concentration exceeding 400 pg/dL. Secondary endpoints included the ratio and difference between baseline and peak NT-proBNP levels. *Result*: Baseline clinical characteristics were well balanced among groups. Over a median follow-up of 399 (252–589) days, HF relapse occurred more frequently in patients who discontinued both S/V and RASB compared to those who maintained either treatment (53.8% vs. 16.3% vs. 10.6%; *p* = 0.001). NT-proBNP levels also showed a more pronounced increase in this group. However, there were no significant differences in primary or secondary outcomes between the S/V and RASB groups. *Conclusions*: Our findings suggest that replacing S/V with another RASB does not worsen outcomes in patients with HFimpEF after S/V treatment, whereas discontinuation of both therapies is associated with a significantly higher risk of HF relapse. A prospective trial is warranted to confirm the safety and effectiveness of this approach in maintaining remission.

## 1. Introduction

Medical therapy for heart failure with reduced ejection fraction (HFrEF) has substantially improved outcomes [1], and a significant proportion of patients experience recovery of left ventricular ejection fraction (LVEF), sometimes approaching normal levels [2,3,4]. As this population of patients with improved LVEF continues to grow, questions have emerged regarding their optimal long-term pharmacological management [5,6]. Prior studies have shown that complete withdrawal of heart failure medications may lead to relapse [3].

These findings suggest the importance of continuing heart failure therapy even after recovery of LVEF. However, the question of how to best maintain therapy in this population remains unanswered. In particular, data are limited regarding the impact of continuing versus tapering or specific medications, such as sacubitril/valsartan (S/V), in patients with heart failure with improved ejection fraction (HFimpEF). One small retrospective study suggested a potential association between dose tapering or switching to renin–angiotensin system blockers (RASBs) and deterioration in LVEF. However, its interpretation is limited by the small sample size and potential confounding factors, such as baseline imbalances and adherence issues [2].

Therefore, we aimed to investigate whether withdrawal of S/V with or without replacement with an RASB while maintaining other guideline-recommended HF medication is associated with HF relapse.

## 2. Material and Methods

### 2.1. Study Population

**Figure 1:**
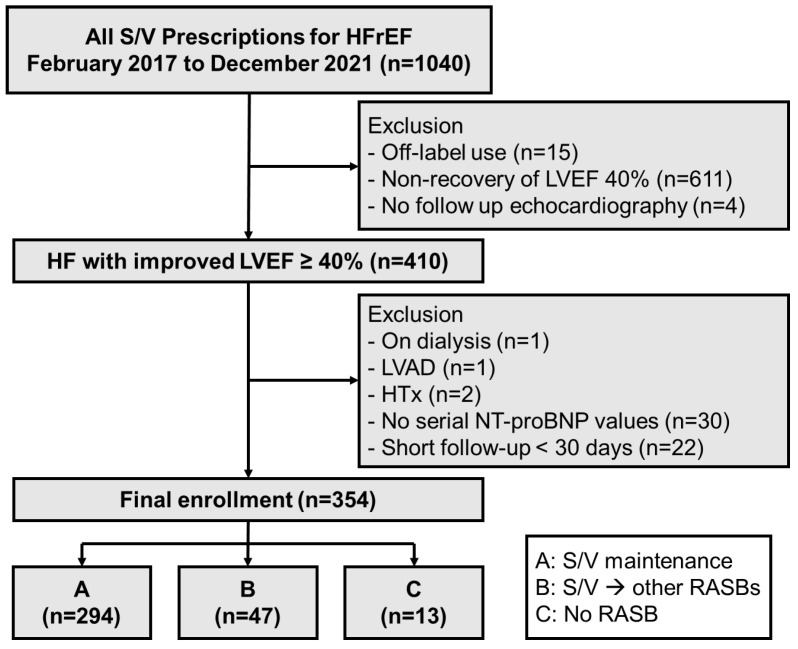
Study flowchart. HFrEF = heart failure with reduced ejection fraction (<40%); S/V = sacubitril/valsartan; LVEF = left ventricular ejection fraction; LVAD = left ventricular assist device; HT = heart transplantation; NT-proBNP = N-terminal-pro hormone B-type natriuretic peptide; RASB = renin–angiotensin system blocker.

This was a single-center retrospective cohort study. Inclusion criteria were patients older than 18 years with HFrEF who were treated with S/V between February 2017 and December 2021 from Samsung Medical Center (Seoul, Republic of Korea). Exclusion criteria comprised off-label prescription, failure to recover LVEF to at least 40%, and lack of echocardiography since starting S/V (Figure 1). Among 410 patients who showed an improvement in LVEF to 40 percent or higher at least once after initiation of S/V, those on dialysis, those who underwent left ventricular assist device (LVAD) implantation or heart transplantation, and those with a short follow-up duration (<30 days) or without serial NT-proBNP measurements were excluded. The threshold of an LVEF of 40% higher to define improvement was based on recent international guidelines for HFimpEF [7].

All boards waived the requirement for informed consent as patients were retrospectively enrolled and data were collected after being anonymized. Our study was conducted in accordance with the Declaration of Helsinki and Institutional Review Board (IRB) approval was obtained (IRB No. 2020-08-082). Neither patients nor the public were involved in any aspect of this study, including its design, conduct, reporting, or dissemination plans.

### 2.2. Definitions and Outcomes

**Figure 2:**
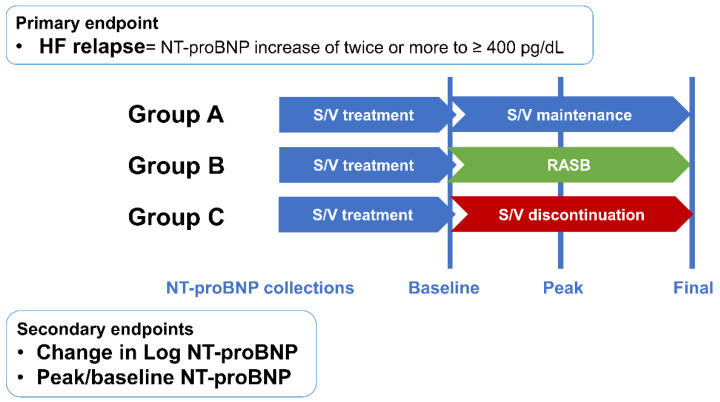
Study endpoints. HF = heart failure; NT-proBNP = N-terminal-pro hormone B-type natriuretic peptide; S/V = sacubitril/valsartan; RASB = renin–angiotensin system blocker.

Patients were divided into three groups according to the use of S/V and RASB after improvement of LVEF (group A: S/V maintenance; group B: change to RASB; group C: neither S/V nor RASB; Figure 1). Patients’ clinical characteristics, medical history, and laboratory and echocardiographic data were collected from electronic medical records from the time of first improved LVEF to at least 40% for group A and for time to S/V discontinuation in groups B and C (Figure 2). Serum NT-proBNP levels were collected at three points: baseline, peak, and final levels. Peak levels were defined as the first value that met the primary endpoint or the highest value during follow-up, as appropriate. Final values were collected at the most recent follow-up.

The primary endpoint was HF relapse, defined as a two-fold increase in baseline NT-proBNP concentration exceeding 400 pg/dL. Secondary endpoints were peak NT-proBNP level, the difference between baseline and peak NT-proBNP, hospitalization for HF, and heart transplantation or death. Although not pre-specified, a composite outcome was evaluated to enhance clinical relevance. This included a doubling of NT-proBNP to above 400 ng/L, an absolute reduction in LVEF by 10% to below 40%, a 10% or greater increase in LVEDV beyond the normal range, or hospitalization for heart failure, based on criteria used in the TRED-HF trial.

### 2.3. Statistical Analysis

Categorical variables are expressed as percent (frequency) and continuous variables as mean ± standard deviation (SD). Comparison of continuous variables between groups used one-way ANOVA with Tukey’s HSD post hoc analysis or the Kruskal–Wallis H test with Dunn test post hoc analysis. Categorical variables were compared by Chi-square tests with Bonferroni’s correction. The Kaplan–Meier method and log-rank test were used for time-to-event analysis. Clinical outcomes were compared between groups using a Cox proportional hazards regression model to calculate the hazard ratio (HR) and 95% confidence interval (CI). Adjusted HRs and 95% CIs were obtained using Cox regression based on age, estimated glomerular filtration rate (GFR), final S/V dose, duration of S/V and sodium-glucose cotransporter-2 (SGLT-2) inhibitor use (Model 1). Additionally, Model 2 included follow-up β-blocker use as an additional covariate. To evaluate whether the treatment effect differed by baseline characteristics, we conducted interaction analyses between treatment groups and key variables including baseline NT-proBNP tertiles and HF etiology. A paired *t*-test and Wilcoxon test were used for paired continuous variable comparison between baseline and follow-up. The McNemar test was used for paired categorical variable comparison between baseline and follow-up.

Statistical significance was considered at a *p* value < 0.05. Statistical analysis was performed using R Statistical Software (version 4.1.0; R Foundation for Statistical Computing, Vienna, Austria).

## 3. Results

### 3.1. Baseline Characteristics

Among 1040 patients with HFrEF treated with S/V, 410 (39.4%) achieved recovery of LVEF to at least 40% (Figure 1). Among the finally enrolled 354 patients, 294 (83.1%) were maintained on S/V (group A) and 60 (16.9%) patients were either switched to other RASBs (group B, *n* = 47, 13.3%) or taken off the medication (group C, *n* = 13, 3.7%).

Baseline clinical characteristics and echocardiographic and medical treatment of the study population are shown in Table 1. The study population was predominantly male (*n* = 256, 72.3%), and most patients experienced tolerable symptoms of New York Heart Association Fc I or II (44.9% and 49.7%, respectively). The most common cause of HF was dilated cardiomyopathy (48.6%), followed by ischemic heart disease (31.9%). Clinical characteristics were well balanced between group A and B, while group C had shorter stature and lower weight compared to the other groups. Echocardiographic parameters showed that group C had a smaller LV chamber diameter and a higher LVEF at baseline compared to groups A and B. Other than a lower sodium level in group C, there was no difference in baseline laboratory values including NT-proBNP between the three groups. Group A had been receiving the highest dose of S/V over the longest period before this study, while the dose and duration of S/V were similar in groups B and C. The prescription rate of sodium-glucose cotransporter-2 inhibitors was significantly lower in group B than in the other two groups. Prescription of beta blocker or spironolactone was noted in 91.0% and 80.8%, respectively, of the total study population.

Reasons for discontinuation of S/V in groups B and C were recovered LVEF, orthostatic hypotension, other intolerance, and chronic renal disease in that decremental order (Supplementary Table S1). The reason for S/V discontinuation did not differ statistically between these two groups, but there were more patients with orthostatic hypotension and intolerance to S/V in group B, while the majority of group C stopped S/V mainly because of LVEF recovery. In group B, most patients were switched from S/V to valsartan, candesartan, or losartan.

### 3.2. HF Relapse

The mean follow-up duration was 399.0 [252.0–589.0] days in the study population, without difference between groups. The incidence of HF relapse based on NT-proBNP levels was significantly higher in group C compared to A and B (53.8% vs. 16.3% vs. 10.6%, *p* = 0.001; Table 2).

**Figure 3:**
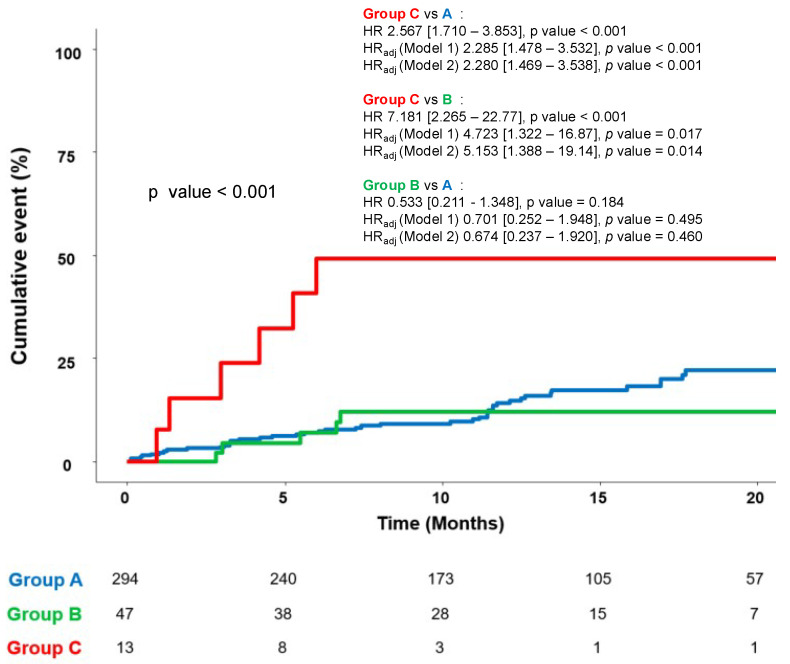
Cumulative incidence of primary endpoint up to 20 months. Model 1: Adjusted for age, estimated GFR, S/V final dose, duration of S/V, and SGLT-2 inhibitor. Model 2: Adjusted for age, estimated GFR, S/V final dose, duration of S/V, SGLT-2 inhibitor and b-blocker use at follow-up. (A = S/V group; B = RASB group; C = group without RASB). GFR = glomerular filtration rate; SGLT-2 = sodium-glucose co-transporter 2; S/V = sacubitril/valsartan; RASB = renin–angiotensin system blocker.

In the Cox regression analysis, the HR for HF relapse in group C was at least four times higher than that of group A (HR_adj_ C to A 2.285, 95% CI [1.478–3.532], *p* < 0.001) or group B (HR_adj_ C to B 4.723, 95% CI [1.322–16.87], *p* = 0.017). There was no significant difference in HF relapse rates between group A and group B (HR_adj_ B to A 0.701, 95% CI [0.252–1.948], *p* = 0.495) (Figure 3). The HR was calculated after adjusting for baseline variables—age, eGFR, final S/V dose, duration of S/V treatment, and SGLT-2 inhibitor usage—which were significantly different and clinically meaningful (Model 1). The results were unchanged after further adjustment (Model 2) for follow-up β-blocker use.

In the Cox proportional hazards model evaluating the primary outcome across baseline NT-proBNP tertiles, there was no statistically significant interaction between treatment group (group A vs. group B) and NT-proBNP tertile (interaction term: HR = 0.774, 95% CI: 0.361–1.661, *p* = 0.511). Similarly, to evaluate whether the treatment effect differed by underlying etiology, we conducted interaction analyses between the three-group treatment variable (groups A, B, and C) and heart failure etiology (dilated cardiomyopathy or ischemic heart failure). In both models, the interaction terms were not statistically significant (group × dilated cardiomyopathy: HR 0.758, 95% CI [0.259–2.221], *p* = 0.614; group × ischemic heart failure: HR 0.617, 95% CI [0.204–1.860], *p* = 0.391), indicating that the effect of treatment strategy on HF relapse did not differ significantly across etiologic subgroups.

### 3.3. Secondary Endpoints and Composite Outcome

**Figure 4:**
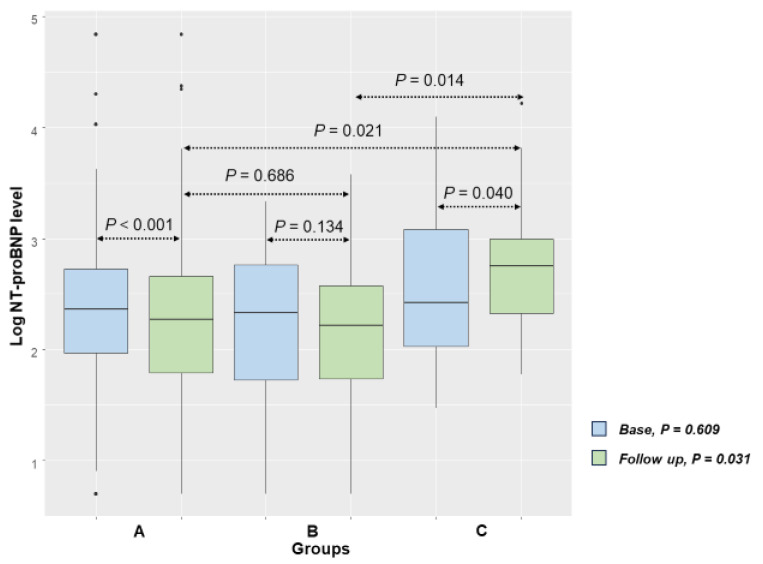
Baseline and follow-up Log NT-proBNP values (A = S/V group; B = RASB group; C = group without RASB).

When examining peak and base NT-proBNP levels, no significant difference was observed in group A and group B. However, group C showed a significant increase in NT-proBNP levels compared to baseline (*p* = 0.002; Table 2). Peak and follow-up levels of serum NT-proBNP were also higher in group C (Table 2). The differences between baseline and follow-up NT-proBNP levels are depicted in Figure 4. While similar at baseline, there was an eventual difference between groups. Group C had higher NT-proBNP levels compared to groups A (*p* = 0.021) and B (*p* = 0.014) at follow-up. A comparison of baseline and follow-up NT-proBNP levels revealed that group B exhibited no statistically significant change (*p* = 0.134), whereas group A showed a significant decrease (*p* < 0.001) and group C demonstrated a significant increase (*p* = 0.040).

Clinical events of hospitalization for HF, heart transplantation, and mortality occurred in only a small number of patients, and there were no significant differences among the three groups (Table 3). Among the five patients who died, there were no cases of cardiac death.

Consistent with the primary outcome, there was no statistically significant difference in composite event rates between groups A and B. However, group C showed a higher incidence of composite events compared to both groups A and B (Supplement Figure S1). This finding remained consistent after adjustment for age, eGFR, final S/V dose, duration of S/V treatment, and SGLT-2 inhibitor usage, as well as when adjusting for follow-up β-blocker use.

### 3.4. Change During Follow-Up Period

In the follow-up data of the three groups with medication change after LVEF improvement, several changes compared to baseline were observed. First, all groups showed a numerical increase in both systolic and diastolic blood pressure. However, only in group C was the increase in diastolic blood pressure significant (diastolic blood pressure: 60.5 mmHg to 69.2 mmHg). Heart rate showed no significant difference between baseline and follow-up in either group A or B, but a significant increase was observed in group C (heart rate 73.5 bpm to 94.0 bpm). Second, the LV end-diastolic dimension decreased significantly in both group A and group B compared to baseline but showed no notable difference in group C. The LV dimension and LV mass index decreased significantly in both group A and group B compared to baseline, whereas they increased in group C, but not significantly. LVEF significantly improved in group A compared to baseline (LVEF 46.6% to 51.6%), increased in group B but without statistical significance (LVEF 48.3% to 56.0%), and decreased in group C (LVEF 55.0% to 51.1%). Third, no significant changes in laboratory results were observed from baseline through follow-up (Table 4).

In the overall cohort, the prescription rate of β-blockers significantly decreased from 91% to 59.6%, while that of SGLT-2 inhibitors significantly increased from 37.3% to 48.3%. In group A, β-blocker use declined, whereas SGLT-2 inhibitor prescription significantly increased, with no notable change in spironolactone use. In group B, β-blocker and spironolactone prescriptions significantly decreased, while SGLT-2 inhibitor use significantly increased. In group C, β-blocker use showed a numerical decrease without statistical significance, and SGLT-2 inhibitor use also increased numerically but was not statistically significant (Table 5).

## 4. Discussion

While S/V has consistently demonstrated superior clinical outcomes compared to other RASBs, there is a paucity of data directly comparing its efficacy to other agents in patients with improved heart failure [8,9,10]. Given its known superior anti-fibrotic effects compared to valsartan, this study is meaningful in that it evaluates the clinical impact of subsequent therapy among patients who had been well stabilized on S/V [11].

The main finding of this study is the significantly increased incidence of HF relapse, defined as a two-fold increase in serum NT-proBNP level to higher than 400 pg/dL, in patients with HF with S/V discontinuation after LVEF improvement (group C); the change was not different between patients who maintained S/V or changed to another RASB (groups A and B). The difference between peak and baseline log NT-proBNP levels showed an interval increase in group C in contrast to an interval decrease in groups A and B.

### 4.1. Cardiac Function Recovery in the S/V Era

The reported proportion of patients who achieved recovery of LVEF varies across studies [2,5,6,12,13]. This variability could be due to the variable LVEF of enrolled patients, definition of recovered HF, and/or prescribed medication. From our cohort of all patients with HFrEF treated with S/V, 39.4% (*n* = 410 of 1040) experienced LVEF recovery to at least 40%. Recovery of cardiac function is an important indicator in the treatment of HF, as it is associated with improved prognosis [14]. Although previous studies have confirmed that the use of S/V leads to better outcomes compared to other RASBs, data on the rate of LVEF improvement remain limited [15,16]. However, the current study is limited by its retrospective design, and further confirmation through prospective studies with large numbers of patients is necessary.

### 4.2. Maintenance Strategy in HFimpEF

There are limited data on outcomes after tapering HF medication in HFimpEF. The well-known TRED-HF trial resulted in a 40% relapse of HF rate six months after withdrawing all HF medication including angiotensin-converting enzyme inhibitor and angiotensin II receptor blocker, beta blocker, mineralocorticoid receptor antagonist, and loop diuretics [3]. Other studies that investigated the effect of tapering an HF medication other than RASB all reported clinical deterioration [17,18,19]. In one of these studies, the effect of the medication being tapered itself was also tested by protocol-specified withdrawal after double-blinded, randomized treatment with empagliflozin versus placebo. The result was exacerbated HF after withdrawal from empagliflozin but not from placebo [17].

S/V reduces NT-proBNP levels in HF across the LVEF spectrum [20,21,22]. Various data have reported S/V to be associated with a greater NT-proBNP reduction compared to other RASBs in HF patients [22,23]. However, the benefit of maintaining S/V in patients with HFimpEF has not been investigated. In a retrospective analysis by Chang et al., patients who had achieved LVEF recovery to 50 percent or higher after treatment with S/V experienced a decrease in LVEF and an increased incidence of the composite outcome of cardiovascular death and heart failure hospitalization over 18 months when the dose of S/V was tapered or replaced with an RASB, compared to those who maintained the same S/V dose alongside other HF medications such as beta-blockers and MRA [2]. However, patients with recovered LVEF were not the primary analysis group, and the number of subjects was small. Therefore, to the best of our knowledge, we are the first to report that clinical outcomes are comparable between patients maintaining S/V and those switching to an RASB after successful improvement of LVEF in patients with HFrEF, while the outcomes are worse in those who discontinued S/V and did not start an RASB.

The clinical conditions associated with the primary endpoint were varied. A recent analysis of the TRED-HF trial reported that lower heart rate was associated with a lower risk of relapse in patients withdrawn from HF medication [24]. In this study, the C group exhibited a higher relapse rate and a significantly higher heart rate at follow-up compared to the other groups (94.0 [82.0–100.0] bpm in group C). Although statistically non-significant, it was accompanied by a decrease in LVEF from 55% to 51.1%. These findings suggest that discontinuing S/V may affect hemodynamics by triggering an increase in sympathetic tone and reactivation of the renin–angiotensin system. It is also noteworthy that the primary outcomes in group C, the medication discontinuation group, occurred predominantly within six months, similar to the TRED-HF study. However, due to the retrospective nature of this study, we were unable to directly assess these hemodynamic changes using advanced imaging or neurohormonal markers.

Chang et al. reported increased LVEF and less deterioration over six months in the group that maintained S/V compared to the tapered group (LVEF 56.4 ± 5.3% vs. 45.0 ± 12.8%, *p* < 0.001; ΔLVEF 1.2 ± 5.1% vs. −9.3 ± 12.0%, *p* < 0.001) [2]. In contrast, our study showed improvements in LV dimensions and LVEF in both group A and group B. This discrepancy may be attributed to differences in reimbursement policies between the two countries, which could have influenced patient selection in the tapering group of Chang et al.’s study. These policies may have resulted in a patient population with lower economic status, differing from that of our cohort.

HFimpEF patients are known to have substantially lower incidence of cardiovascular events and hospitalizations compared to patients with HFrEF and HF with preserved EF [5]. The TRED-HF trial reported no events within the initial six month study period [3]. Chang et al. reported more frequent hospitalization for HF and cardiovascular death in the tapered group than the maintenance group (23.1% vs. 5.9%, HR 0.22, *p* = 0.035) [2]. Although our study had a longer observation period of 13 months, the clinical event rates were too low to discriminate between groups. A long-term study is needed to determine whether tapering S/V will result in increased cardiovascular events.

### 4.3. Interpretation of the Primary Endpoint

For those who met the primary endpoint, the clinical conditions associated with an increase in NT-proBNP increase are shown in Supplementary Table S2. Among patients with elevated NT-proBNP levels, 35% of patients did not exhibit any distinct clinical symptoms. Approximately 20% of patients who met the primary endpoint exhibited signs indicative of cardiac function deterioration, such as tachyarrhythmia, acute cardiovascular events, or progressive LV failure. Cases that could have demonstrated non-cardiac elevations in NT-proBNP, such as infection, renal dysfunction, and anemia, accounted for 25% of the study population.

### 4.4. Limitations

This study needs to be interpreted with caution due to several limitations. First, due to its retrospective design, baseline time points had to be different between groups who maintained or discontinued S/V. However, the average time difference between LVEF improvement and S/V discontinuation in groups B and C was only three days, hindering conclusion of baseline differences. In addition, as with any retrospective design, it was not possible to capture all symptom changes beyond what was documented in the medical records. Second, this was a small single-center study with a short observation period of approximately one year. Since this study was conducted at a referral center, there is a possibility that patients with relatively greater severity were included. Furthermore, since all participants were of East Asian ethnicity and the majority were male, the generalizability of the findings may be limited. Third, follow-up echocardiographic data were not available in all patients. The absence of these data may limit the ability to detect subclinical remodeling and impose limitations on the interpretation of the results. Additionally, although socioeconomic factors could have influenced medication adherence, relevant data were not available for analysis, which represents another limitation of the study. However, in Korea, S/V is reimbursed for patients with a prior history of HFrEF, even after improvement in LVEF. Fourth, the prescription rate of SGLT-2 inhibitors is lower than that of other medications, as they have been officially recognized as a treatment for heart failure only since 2021, with insurance coverage in Korea beginning after 2024. However, due to these changes, the prescription of SGLT-2 inhibitors increased during follow-up compared to baseline. Fifth, although it would be ideal to assess the effect of ARNI tapering in a setting without changes to other medications, this was not the case in our study. During the follow-up period, β-blocker use declined across all groups, while SGLT2 inhibitor use increased. In particular, group C showed a marked decrease in β-blocker prescription, which may have acted as a confounding factor. Finally, this study enrolled clinically stable patients who had received S/V at adequate doses for approximately one year and had a relatively low baseline NT-proBNP level (in the mid-200 pg/mL range on average). This may have introduced selection bias; thus, caution is warranted when generalizing these findings to patients with shorter exposure to S/V or those with persistently elevated NT-proBNP levels.

Despite these limitations, the lack of differences in clinical outcomes and hemodynamics between the group that continued S/V and the group that switched to other RASBs in patients with improved HF is a novel finding not observed in previous studies. To confirm this result, studies with larger numbers of participants and a prospective design are needed. In line with this necessity, we are currently conducting a prospectively designed study (NCT04803175) to overcome this limitation.

## 5. Conclusions

In a retrospective analysis from a single tertiary hospital, replacing S/V with another RASB did not result in worse outcomes in HF patients who had achieved an improved LVEF of at least 40% and resolved the symptoms. A prospective trial is needed to determine whether S/V replacement with other RASBs is safe and effective in maintaining remission after improvement of LVEF in HFrEF patients.

## Figures and Tables

**Table 1:** Baseline characteristics.

	Total (*n* = 354)	A (*n* = 294)	B (*n* = 47)	C (*n* = 13)	*p*-Value	Post-Hoc
Male	256 (72.3%)	212 (72.1%)	37 (78.7%)	7 (53.8%)	0.203	
Age, yr	63.0 [51.0–72.0]	62.5 [51.0–72.0]	64.0 [43.5–68.0]	68.0 [57.0–76.0]	0.179	
Height, cm	166.0 [158.0–172.0]	166.0 [158.0–172.0]	169.0 [159.0–174.5]	157.5 [148.0–167.0]	**0.018**	A = B > C
Weight, kg	69.9 [59.9–79.0]	69.9 [60.3–79.0]	72.5 [60.0–86.2]	56.0 [50.5–69.0]	**0.009**	A = B > C
BSA, kg/m^2^	1.8 ± 0.2	1.8 ± 0.2	1.8 ± 0.3	1.6 ± 0.2	**0.009**	A = B > C
NYHA Fc					0.136	
I	159 (44.9%)	138 (46.9%)	20 (42.6%)	1 (7.7%)		
II	176 (49.7%)	142 (48.0%)	24 (51.1%)	10 (76.9%)		
III	18 (5.1%)	13 (4.4%)	3 (6.4%)	2 (15.4%)		
**Heart failure etiology**						
Dilated cardiomyopathy	172 (48.6%)	148 (50.3%)	21 (44.7%)	3 (23.1%)	0.133	
Ischemic heart failure	113 (31.9%)	96 (32.7%)	14 (29.8%)	3 (23.1%)	0.694	
New onset heart failure	116 (32.8%)	96 (32.7%)	16 (34.0%)	4 (30.8%)	0.975	
**Comorbidities**						
Hypertension	162 (45.8%)	140 (47.6%)	18 (38.3%)	4 (30.8%)	0.266	
Previous coronary revascularization	112 (31.6%)	94 (32.0%)	15 (31.9%)	3 (23.1%)	0.791	
Diabetes mellitus	115 (32.5%)	93 (31.6%)	15 (31.9%)	7 (53.8%)	0.249	
Previous myocardial infarction	52 (14.7%)	44 (15.0%)	7 (14.9%)	1 (7.7%)	0.758	
Coronary artery disease	99 (28.0%)	85 (28.9%)	11 (23.4%)	3 (23.1%)	0.667	
Atrial fibrillation/flutter	86 (24.3%)	70 (23.8%)	11 (23.4%)	5 (38.5%)	0.486	
**Echocardiography**						
LV end-diastolic dimension, mm	55.5 [51.3–58.9]	55.6 [52.0–59.3]	55.7 [51.6–57.6]	47.1 [43.6–48.3]	**<0.001**	A = B > C
LVEF, %	47.0 [43.0–55.0]	46.4 [42.6–53.7]	48.3 [44.5–57.3]	55.0 [47.3–58.0]	**0.022**	A = B < C
**Laboratory finding**						
Hemoglobin, g/dL	13.7 [12.1–15.0]	13.7 [12.3–15.0]	13.6 [12.0–14.9]	11.5 [10.6–13.7]	0.055	
Sodium, mmol/L	140.0 [138.0–141.0]	140.0 [138.0–141.0]	140.0 [138.5–141.0]	138.0 [134.0–140.0]	**0.022**	A = B > C
NT-proBNP, pg/dL	230.0 [87.3–536.0]	233.5 [92.9–525.0]	214.0 [54.6–584.5]	265.0 [106.0–1215.0]	0.603	
**Baseline treatment**						
S/V dose, mg	200.0 [100.0–400.0]	200.0 [100.0–400.0]	100.0 [100.0–200.0]	100.0 [100.0–100.0]	**<0.001**	A > B = C
S/V duration before enrollment, days	436.0 [210.0–660.0]	486.5 [290.5–700.0]	120.0 [64.5–306.5]	219.0 [126.0–297.0]	**<0.001**	A > B = C
Beta blocker	322 (91.0%)	270 (91.8%)	42 (89.4%)	10 (76.9%)	0.171	
Spironolactone	286 (80.8%)	235 (79.9%)	42 (89.4%)	9 (69.2%)	0.175	
SGLT-2 inhibitor	132 (37.3%)	119 (40.5%)	9 (19.1%)	4 (30.8%)	**0.017**	A = C > B
Loop diuretics	151 (42.7%)	124 (42.2%)	20 (42.6%)	7 (53.8%)	0.707	
Previous CRT	22 (6.2%)	18 (6.1%)	2 (4.3%)	2 (15.4%)	0.335	
Previous ICD	28 (7.9%)	26 (8.8%)	1 (2.1%)	1 (7.7%)	0.285	

ACEI = angiotensin-converting enzyme inhibitor; ARB = angiotensin II receptor blocker; BSA = body surface area; BUN = blood urea nitrogen; CRT = cardiac resynchronization therapy; DCMP = dilated cardiomyopathy; EF = ejection fraction; GFR = glomerular filtration rate; ICD = implantable cardioverter-defibrillator; LV = left ventricle/ventricular; LAVI = left atrial volume index; NYHA Fc = New York Heart Association functional class; RV = right ventricle/ventricular; SGLT-2 inhibitor = sodium-glucose cotransporter-2; S/V = sacubitril/valsartan. Data are presented as mean ± standard deviation, median [25th percentile–75th percentile], or *n* (%). The values in bold indicate statistical significance (*p* < 0.05).

**Table 2:** Primary and secondary endpoints.

	A (*n* = 294)	B (*n* = 47)	C (*n* = 13)	*p*-Value	Post Hoc
Primary endpoint *	48 (16.3%)	5 (10.6%)	7 (53.8%)	**0.001**	A = B < C
Ratio of NT-proBNP (Peak/Base)	1.09 [0.79–1.80]	1.10 [0.89–1.75]	2.52 [1.90–5.66]	**0.002**	A = B < C
Change in log (Peak − Base)	0.04 [−0.10–0.26]	0.04 [−0.05–0.24]	0.40 [0.28–0.75]	**0.002**	A = B < C
Baseline NT-proBNP	233.5 [92.9–525.0]	214.0 [54.6–584.5]	265.0 [106.0–1215.0]	0.603	
Peak NT-proBNP	259.5 [103.0–676.0]	246.0 [100.0–610.8]	888.0 [560.0–2305.0]	**0.014**	A = B < C
Follow-up NT-proBNP	188.0 [61.7–463.0]	163.0 [54.0–376.5]	568.0 [210.0–992.0]	**0.031**	A = B < C
Baseline Log NT-proBNP	2.4 [2.0–2.7]	2.3 [1.7–2.8]	2.4 [2.0–3.1]	0.603	
Peak Log NT-proBNP	2.4 [2.0–2.8]	2.4 [2.0–2.8]	2.9 [2.7–3.4]	**0.014**	A = B < C
Follow-up Log NT-proBNP	2.3 [1.8–2.7]	2.2 [1.7–2.6]	2.8 [2.3–3.0]	**0.031**	A = B < C

Units for all NT-proBNP levels, pg/dL. NT-proBNP—N-terminal-pro hormone B-type natriuretic peptide. * Primary endpoint was HF relapse defined as a two-fold increase in baseline NT-proBNP concentration to greater than 400 pg/dL. Ratio of NT-proBNP = Peak NT-proBNP/Base NT-proBNP. Data are presented as median [25th percentile–75th percentile] or *n* (%). The values in bold indicate statistical significance (*p* < 0.05).

**Table 3:** Clinical outcomes.

*N* (%) or Mean ± SD	Total (*n* = 354)	A (*n* = 294)	B (*n* = 47)	C (*n* = 13)	*p*-Value
Follow-up duration, days	399 [252–589]	397 [241–596]	440 [332–574]	300 [273–340]	0.062
Hospitalization for heart failure	3 (0.8%)	3 (1.0%)	0 (0.0%)	0 (0.0%)	0.734
Heart transplantation	1 (0.3%)	1 (0.3%)	0 (0.0%)	0 (0.0%)	0.903
Mortality	5 (1.4%)	4 (1.4%)	0 (0.0%)	1 (7.7%)	0.113

Data are presented as median [25th percentile–75th percentile] or *n* (%).

**Table 4:** Vital signs, echocardiography, and laboratory result changes during follow-up.

	Total (*n* = 354)	A (*n* = 294)	B (*n* = 47)	C (*n* = 13)	*p*-Value	Post Hoc
**Vital signs at baseline (*n* = 351)**						
Systolic blood pressure, mmHg	113.0 [102.0–127.0]	113.0 [102.0–128.0]	113.0 [104.0–126.0]	104.5 [96.5–123.0]	0.456	
Diastolic blood pressure, mmHg	63.2 ± 14.6	63.4 ± 14.9	62.8 ± 13.9	60.5 ± 8.9	0.772	
Heat rate (bpm)	74.0 [67.0–83.0]	74.0 [66.0–83.0]	78.0 [71.0–84.0]	73.5 [71.0–83.0]	0.168	
**Vital signs at follow-up (*n* = 297)**						
Systolic blood pressure, mmHg	116.0 [104.0–131.0]	116.0 [104.5–130.0]	118.0 [106.0–134.0]	106.0 [101.0–132.0]	0.702	
Diastolic blood pressure, mmHg	66.0 ± 14.9	66.0 ± 14.9	65.3 ± 14.5	69.2 ± 17.2 *	0.699	
Heart rate (bpm)	75.0 [67.0–85.0]	74.0 [65.0–83.0]	78.0 [70.0–84.0]	94.0 [82.0–100.0]	0.001	A = B < C
**Echocardiography at baseline (*n* = 354)**						
LV end-diastolic dimension, mm	55.5 [51.3–58.9]	55.6 [52.0–59.3]	55.7 [51.6–57.6]	47.1 [43.6–48.3]	**<0.001**	A = B > C
LV end-systolic dimension, mm	38.6 [33.2–43.2]	38.8 [34.2–43.3]	37.2 [32.9–43.5]	30.1 [29.4–32.1]	**0.001**	A = B > C
LV mass index, g/m^2^	106.7 [92.1–125.0]	107.2 [92.1–126.1]	103.8 [93.4–121.9]	95.1 [86.1–118.1]	0.632	
LVEF, %	47.0 [43.0–55.0]	46.4 [42.6–53.7]	48.3 [44.5–57.3]	55.0 [47.3–58.0]	**0.022**	A = B < C
LAVI, mL/m^2^	36.9 [29.1–48.5]	36.8 [28.9–48.9]	37.4 [31.6–43.7]	46.5 [37.8–53.1]	0.413	
E/e’	10.0 [7.8–13.3]	9.8 [ 7.6–13.6]	9.8 [ 7.6–13.6]	11.9 [ 7.0–15.0]	0.881	
RV systolic pressure, mmHg	26.4 [23.3–30.0]	26.4 [23.4–29.2]	26.4 [23.4–29.2]	26.2 [25.5–33.1]	0.676	
**Echocardiography at follow-up (*n* = 297)**						
LV end-diastolic dimension, mm	54.0 [50.0–57.5] *	54.2 [50.3–58.0] *	53.4 [50.4–57.0] *	48.3 [46.3–52.1]	**0.047**	A = B > C
LV end-systolic dimension, mm	36.0 [32.2–41.2] *	36.8 [33.0–41.3] *	34.0 [31.9–40.6] *	31.0 [28.9–35.9]	0.090	
LV mass index, g/m^2^	99.2 [85.2–115.9] *	99.4 [84.8–117.5] *	98.8 [87.4–113.2] *	89.6 [87.7–107.7]	0.786	
LVEF, %	52.5 [45.4–58.1] *	51.6 [44.9–58.0] *	56.0 [48.6–59.0]	51.1 [51.0–58.5]	0.085	
LAVI, mL/m^2^	36.2 [27.9–46.6]	35.8 [27.9–45.6]	37.5 [28.8–47.8]	34.5 [20.3–58.6]	0.868	
E/e’	9.7 [7.3–13.5]	9.8 [ 7.4–13.5]	8.2 [ 6.8–11.2]	9.7 [ 7.8–17.2]	0.483	
RV systolic pressure, mmHg	25.7 [22.9–31.5]	25.4 [22.9–31.4]	28.6 [24.3–31.5]	23.0 [20.6–31.5]	0.370	
**Laboratory at baseline**						
Hemoglobin, g/dL (*n* = 311)	13.7 [12.1–15.0]	13.7 [12.3–15.0]	13.6 [12.0–14.9]	11.5 [10.6–13.7]	0.055	
BUN, mg/dL (*n* = 347)	17.1 [13.3–21.8]	17.2 [13.2–21.5]	16.3 [13.4–21.4]	25.0 [14.2–45.1]	0.174	
Creatinine, mg/dL (*n* = 346)	0.9 [0.8–1.1]	0.9 [0.8–1.1]	1.0 [0.8–1.2]	1.1 [0.8–1.7]	0.550	
eGFR, mL/min/1.73 m^2^ (*n* = 346)	81.8 [60.3–96.4]	82.4 [60.7–96.8]	81.4 [66.2–93.2]	70.5 [40.5–89.8]	0.347	
Sodium, mmol/L (*n* = 345)	140.0 [138.0–141.0]	140.0 [138.0–141.0]	140.0 [138.5–141.0]	138.0 [134.0–140.0]	**0.022**	A = B > C
Potassium, mg/dL (*n* = 345)	4.4 [4.1–4.8]	4.4 [4.1–4.8]	4.4 [4.1–4.8]	4.4 [4.2–4.9]	0.953	
**Laboratory at follow-up**						
Hemoglobin, g/dL (*n* = 321)	13.8 [12.5–14.7]	13.9 [12.5–14.9]	13.1 [12.5–14.4]	11.8 [10.4–14.6]	0.050	
BUN, mg/dL (*n* = 353)	17.0 [13.2–22.2]	16.8 [13.2–22.2]	17.6 [13.8–21.8]	18.1 [12.8–23.3]	0.958	
Creatinine, mg/dL (*n* = 353)	1.0 [0.8–1.1]	0.9 [0.8–1.1]	1.0 [0.9–1.1]	0.9 [0.6–1.1]	0.690	
eGFR, mL/min/1.73 m^2^ (*n* = 353)	81.0 [62.1–93.7]	81.0 [60.9–94.0]	81.4 [63.7–91.8]	76.9 [61.1–97.2]	0.966	
Sodium, mmol/L (*n* = 353)	140.0 [138.0–141.0]	140.0 [138.0–141.0]	140.0 [138.0–141.0]	139.0 [137.0–141.0]	0.318	
Potassium, mg/dL (*n* = 353)	4.5 [4.2–4.8]	4.5 [4.2–4.8]	4.5 [4.0–4.8]	4.5 [4.4–5.3]	0.242	

BUN, blood urea nitrogen; EF, ejection fraction; eGFR, estimated glomerular filtration rate; LAVI, left atrial volume index; LV, left ventricle/ventricular; RV, right ventricle/ventricular. Data are presented as mean ± standard deviation, median [25th percentile–75th percentile], or *n* (%). The values in bold indicate statistical significance (*p* < 0.05). * *p* < 0.05 compared to baseline.

**Table 5:** Medication changes during follow-up.

	Total (*n* = 354)	A (*n* = 294)	B (*n* = 47)	C (*n* = 13)	*p*-Value
**Medication at baseline**					
Beta blocker	322 (91.0%)	270 (91.8%)	42 (89.4%)	10 (76.9%)	0.171
Spironolactone	286 (80.8%)	235 (79.9%)	42 (89.4%)	9 (69.2%)	0.175
SGLT-2 inhibitor	132 (37.3%)	119 (40.5%)	9 (19.1%)	4 (30.8%)	0.017
**Medication at follow-up**					
Beta blocker	211 (59.6%) *	176 (59.9%) *	30 (63.8%) *	5 (38.5%)	0.250
Spironolactone	274 (77.4%)	231 (78.6%)	34 (72.3%) *	9 (69.2%)	0.493
SGLT-2 inhibitor	171 (48.3%) *	149 (50.7%) *	16 (34.0%) *	6 (46.2%)	0.105

SGLT-2, Sodium–Glucose Cotransporter 2. Data are presented as *n* (%). * *p* < 0.05 compared to baseline.

## Data Availability

Due to patient confidentiality and ethical restrictions, the data are not publicly available. However, deidentified data may be shared upon reasonable request and with approval from the Institutional Review Board.

## Supplementary Materials

The following supporting information can be downloaded at https://www.mdpi.com/article/10.3390/medicina61081487/s1, Table S1: Characteristics of the patients who discontinued S/V. Table S2: Clinical conditions of patients who achieved the primary endpoint. Figure S1: Cumulative incidence of composite endpoint up to 20 months.

## Abbreviations and Acronyms

HFimpEF	heart failure with improved ejection fraction
HFrEF	heart failure with reduced ejection fraction
LVEF	left ventricular ejection fraction
NT-proBNP	N-terminal-pro hormone B-type natriuretic peptide
RASB	renin–angiotensin system blockers
S/V	sacubitril/valsartan
