# Supplementary material for: Optimal Maintenance Strategy for Patients with Improved Left Ventricular Function Following Sacubitril/Valsartan Therapy
Source: Medicina (Kaunas). 2025 Aug 19;61(8):1487. doi: 10.3390/medicina61081487 (PMC12388812; doi:10.3390/medicina61081487)
Supplement: Supplementary file 1 [file medicina-61-01487-s001.zip › medicina-3768164-supplementary.pdf]

Supplementary Table S1 Characteristics of the groups with discontinuation of S/V

Groups	B & C (n=60)	B (n=47)	C (n=13)	p value between B and C
**Reason for discontinuation of S/V**				1.000
Recovered LVEF	24 (40.0%)	16 (34.0%)	8 (61.5%)	
Orthostatic hypotension	19 (31.7%)	17 (36.2%)	2 (15.4%)	
Intolerance	9 (15.0%)	9 (19.1%)	0 (0.0%)	
Chronic renal disease	5 (8.3%)	2 (4.3%)	3 (23.1%)	
Hyperkalemia	3 (5.0%)	3 (6.4%)	0 (0.0%)	
**Change to ARB**				
Valsartan		37 (78.7%)		
Candesartan		6 (12.8%)		
Losartan		3 (6.4%)		
Olmesartan		1 (2.1%)		

S/V = sacubitril/valsartan; LVEF = left ventricular ejection fraction; ARB = angiotensin II receptor blocker.

Data are presented as n (%).

p value calculated using Fisher's exact test comparing the distribution of all listed reasons for S/V discontinuation between Groups B and C as a single categorical variable.

Supplementary Table S2 Clinical condition of patients who met the primary endpoint

Clinical condition	N (%)
No clinical worsening course	21 (35.0)
Infection	9 (15.0)
Atrial tachyarrhythmia	6 (10.0)
Acute cardiovascular event*	4 (6.7)
Renal dysfunction	4 (6.7)
Postoperative state	4 (6.7)
Trauma	4 (6.7)
Progressive LV failure	2 (3.3)
Anemia	2 (3.3)
Progression of chronic disease	2 (3.3)
Poor compliance	2 (3.3)

LV = left ventricular; ADHF = acute deterioration of heart failure.

*Acute cardiovascular events included: Venous thromboembolism and peripheral vascular ischemia

Data are presented as n (%).

SUPPLEMENT FIGURE LEGEND

Supplement Figure S1. Cumulative incidence of composite endpoint up to 20 months.

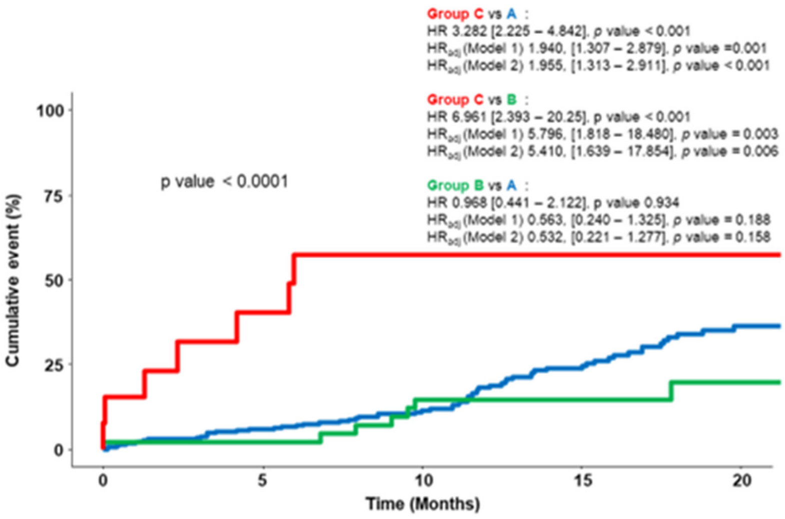

*model 1: Adjusted for age, estimated GFR, S/V final dose, duration of S/V, and SGLT-2 inhibitor

*model 2 : Adjusted for age, estimated GFR, S/V final dose, duration of S/V, SGLT-2 inhibitor and b-blocker use at follow-up

(A=S/V group; B=RASB group; C=group without RASB)

GFR = glomerular filtration rate; SGLT-2 = sodium-glucose co-transporter 2; S/V = sacubitril/valsartan; RASB = renin-angiotensin-system blocker.
